# Parametric estimation of gyrotactic microorganism hybrid nanofluid flow between the conical gap of spinning disk-cone apparatus

**DOI:** 10.1038/s41598-021-03077-2

**Published:** 2022-01-07

**Authors:** Hussam Alrabaiah, Muhammad Bilal, Muhammad Altaf Khan, Taseer Muhammad, Endris Yimer Legas

**Affiliations:** 1 College of Engineering, Al Ain University, Al Ain, United Arab Emirates; 2 Department of Mathematics, Tafila Technical University, Tafila, Jordan; 3 Department of Mathematics, City University of Science and Information Technology, Peshawar, Pakistan; 4 Institute for Groundwater Studies, Faculty of Natural and Agricultural Sciences, University of the Free State, Bloemfontein, South Africa; 5 Department of Mathematics, College of Sciences, King Khalid University, Abha, 61413 Saudi Arabia; 6 Department of Mathematics, College of Natural Science, Wollo University, Dessie, Ethiopia

**Keywords:** Energy science and technology, Mathematics and computing, Nanoscience and technology

## Abstract

The silver, magnesium oxide and gyrotactic microorganism-based hybrid nanofluid flow inside the conical space between disc and cone is addressed in the perspective of thermal energy stabilization. Different cases have been discussed between the spinning of cone and disc in the same or counter wise directions. The hybrid nanofluid has been synthesized in the presence of silver *Ag* and magnesium oxide *MgO* nanoparticulate. The viscous dissipation and the magnetic field factors are introduced to the modeled equations. The parametric continuation method (PCM) is utilized to numerically handle the modeled problem. Magnesium oxide is chemically made up of Mg^2+^ and O^2-^ ions that are bound by a strong ionic connection and can be made by pyrolyzing Mg(OH)^2^ (magnesium hydroxide) and *MgCO*^*3*^ (magnesium carbonate) at high temperature (700–1500 °C). For metallurgical, biomedical and electrical implementations, it is more efficient. Similarly, silver nanoparticle's antibacterial properties could be employed to control bacterial growth. It has been observed that a circulating disc with a stationary cone can achieve the optimum cooling of the cone-disk apparatus while the outer edge temperature remains fixed. The thermal energy profile remarkably upgraded with the magnetic effect, the addition of nanoparticulate in base fluid and Eckert number.

## Introduction

The analysis illustrates that disk-cone devices have a variety of technical and practical implementations, including Oldroyd-B fluid stability analysis, estimation of fluid viscosity, medical requirement, the cooling system of the conical diffuser, convective propagation of feeding culture and in biomedicine^1^. Turkyilmazoglu et al*.*^2^ revealed the solution for constant an incompressible Newtonian fluid flow over a spinning cone using an analytical strategy. Chamkha and Mudhaf^3^ examined the mass and energy transition over a vertical impermeable cone circulating with an unsteady angular velocity in an ambient fluid in the presence of heat absorption or generation and magnetic reactions. When the angular acceleration of the cone improves, the azimuthal and tangential skin-friction factors, as well as the Sherwood and Nusselt numbers improve. In the context of thermal energy radiation, the phenomenon of magnetic nanoliquid flow across a revolving cone has been considered by Nadeem^4^. According to the results of the investigation, the magnetic parameter M reduces velocity in both secondary and primary directions. Gul et al*.*^5^ reported a 3D Darcy MHD Casson fluid, continuous flow across the gap of spinning disc and cone. It has been discovered that as the Peclet and Lewis numbers improve, the motile density of microbes reduces. Li et al.^6,7^ addressed the viscous fluids flow due to a spinning cone and disk. For greater variable viscosity parameters, temperature and velocity exhibit divergent responses. Both entropy optimization and Bejan number have a comparable influence on the growing values of the thermal conductivity variable. Lv et al*.*^8^ evaluated the Hall current, magnetic field, and heat radiation influenced nanofluids to flow on a revolving disk's surface. The goal of their effort was to improve heat conveyance rates for industrial and engineering applications. Ahmadian et al*.*^9,10^ explored at a 3D computational model for Ag-MgO hybrid nanofluid flow with energy and mass transmission generated by an irregular fluctuating spinning disc. The configuration of a circulating disc is thought to have a favorable effect on thermal energy and velocity transmission.

Hybrid nanofluids are a unique type of fluid that operates well in energy translocation when compared to traditional fluids such as water and oil. When the heat intensity is high enough, nanofluids may be used in a wide range of thermal activities^11,12^. Hybrid nanofluid flow is used in a variety of applications, including heat exchangers, heat pipes, solar energy, manufacturing, automobile sector, air conditioning, generator cooling, nuclear system cooling, electronic cooling, ships, transformer cooling and biomedicine^13^. In this study, we examined the characteristics of silver and magnesium oxide nanostructures using water as a carrier fluid. Because of their distinctive chemical and physical features, Silver nanoparticles (AgNPs) are being used in a wide range of industries, including pharmaceuticals, foodstuff, consumption products, medical services, and manufacturing. Among these optical, thermal and electrical characteristics, as well as strong electrical conductivity and biological qualities^14^. Magnesium Oxide Nanoparticles (MgONPs) are an antimicrobial that is harmless and reasonably cheap to obtain. The United States Drug and Food Administration has approved MgONPs as non-toxic and safe chemicals (21CFR184.1431). Recent advancements in technologies and medicine have resulted in notable discoveries with great promise. MgONPs, for instance, can alleviate heartburn, activate bone healing scaffolds after they have been activated, and function as hyperthermia accelerators in cancer treatment^15^. Anuar et al*.*^16^ evaluated the boundary layer flow and thermal performance of a hybrid nanoliquid including silver and magnesium oxide nanocomposites as it passed through an inclined stretching sheet with buoyant and suction force. The observations show that raising the quantity of Ag-MgO/water nanocrystals in nanofluid decreases the Nusselt number. Dinarvand et al*.*^17^ used multiple approaches to investigate the constant laminar magnetohydrodynamics (MHD) flow of an Ag-MgO/water hybrid nanofluid across a lateral thin needle with heat radiation. Bilal et al.^18,19^ addressed the relative contribution of magnetic and electric hydrodynamic effect on the water-based CNTs and iron oxide hybrid nanofluid flow between two circulating plates. Heat transfer has been assumed to rise as the magnetic field, electric term and Reynolds number improve. Sreedevi et al*.*^20^ computationally evaluated the Ag-water-based radiative nanoliquid with fluid flow and heat transition characteristics within a circular tube with thermal conditions on vertical walls. When 0.05 volume fraction silver nanoparticles are dispersed in water, the heat transference rate improves from 6.3 to 12.4%. Zhao et al*.*^21^ investigated the influence of viscous dissipation on the flow of a hybrid nanofluid across a stretched sheet utilising various nanoparticles to improve the fluid's thermal conductivity. Kumar et al*.*^22^ studied the effect of activation energy on the Darcy–Forchheimer flow of Casson fluid using Graphene oxide and Titanium dioxide nanoparticle suspensions in a porous medium using 50% Ethylene glycol as the base fluid. Khan et al*.*^23^ presented about the Marangoni convection of a hybrid nanofluid made up of two nanoparticles and a base fluid. Some related literature may be found in^24–27^.

Shi et al*.*^28,29^ proposed a mathematical framework to evaluate the bio-convection flow of a magneto-cross nanofluid including gyrotactic microorganisms with transient magnetic flow of Cross nanoliquid through a stretched sheet. Yusuf et al*.*^30^ studied the rate of entropy generation in a bio-convective flow of a MHD Williamson nanoliquid across an inclined convectively heated stretchable plate, taking into account the effects of thermal radiation and chemical reaction. The presence of microorganisms has been shown to help in the stabilisation of suspended nanoparticles via a bioconvection mechanism. Khashi'ie et al*.*^31^ and Wahid et al*.*^32^ numerically investigates the influence of gyrotactic microorganisms in the mixed convection stagnation point flow of Cu-Al2O3/water hybrid nanofluid towards an immovable plate.

Highly nonlinear boundary value concerns that cannot be solved are common in the automotive industry. For many problems that are routinely addressed by other numerical methods, convergence is susceptible to relaxation constants and initial framework. The PCM's objective is to uncover the technique's universal applicability as a viable solution to nonlinear issues^33^. The 3D turbulent flow and heat transmission over the surface of an extensible spinning disc is highlighted by Shuaib et al*.*^34^. The fluid has been studied in the presence of external magnetic strength. Shuaib et al*.*^35^ identified the characteristic of an ionic transitional boundary layer flow across a revolving disc. To find an ionic species, the Poisson's and Nernst-Planck equations were utilized. Wang et al*.*^36^ used a parametric continuation technique to analyze the consistency of complex systems for engineering disciplines. They also investigated the static bifurcation that occurs while solving nonlinear starting value problems with distinct features and developed an algorithm for determining the bifurcation points in detail. Bilal et al*.*^37^ explored the fluctuating Maxwell nanofluid flow around a stretched cylinder guided by suction/injection impact. The resulting system of ODEs was then numerically calculated using the PCM technique, and the results were validated using the Matlab program bvp4c.

The above examinations indicated that no attempt has been made so far to analyze the 3D flow of silver, magnesium oxide and gyrotactic microorganism-based hybrid nanofluid inside the conical space between disc and cone in perspective of thermal energy stabilization. Multiple cases involving the rotation of disc and cone in the same or reverse trajectory have been addressed. The hybrid nanocomposites are formed in the context of silver *Ag* and magnesium oxide MgO nanomaterials. The magnetic field and viscous dissipation components are included in the simulated equations. To numerically address the posed situation, the computational methodology parametric continuation method is used.

## Mathematical formulation

**Figure 1:**
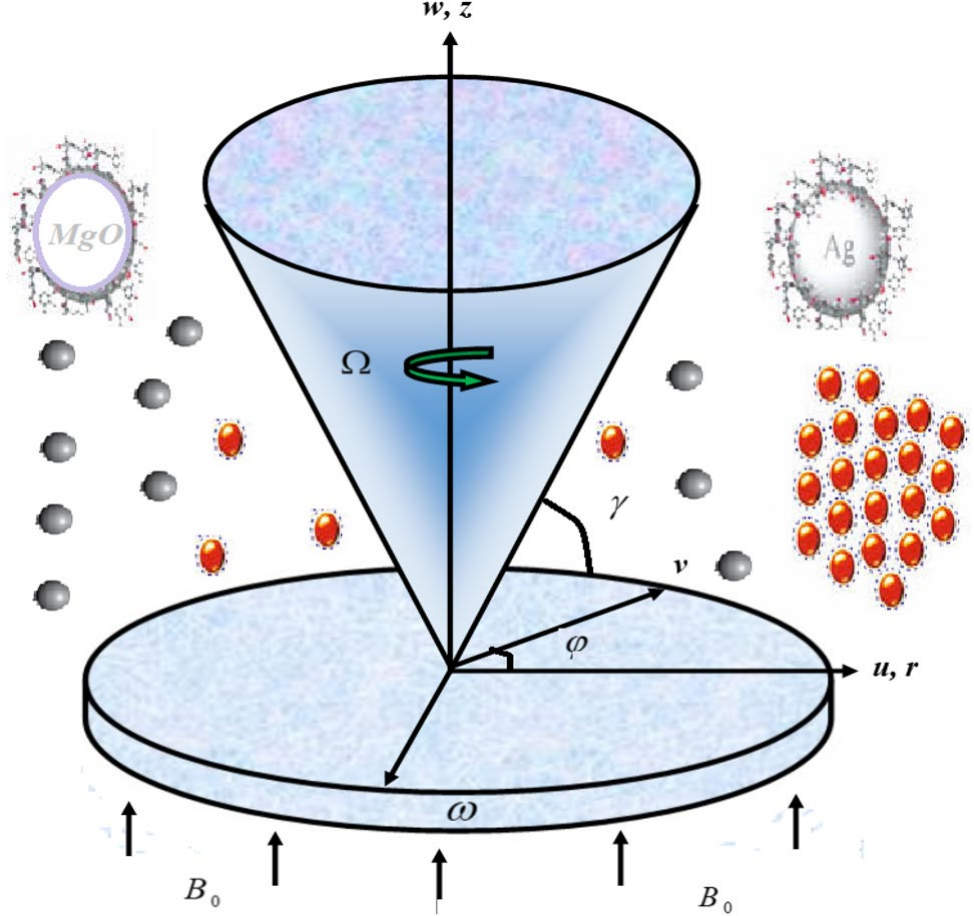
The hybrid nanofluid flow arrangement between the disc and cone.

We considered an incompressible flow of silver and magnesium oxide-based hybrid nanofluid between a cone and disk under the consequences of the magnetic field. The motile microorganism has been also considered in the present analysis. Both the devices are supposed to be either stationary or spinning in the $r,\varphi ,z$ direction (cylindrical coordinate) with the angular velocity. The $\Omega$ and $\omega$ elaborate the cone and disk angular velocities. Figure 1 communicates the hybrid nanofluid flow mechanism between the disk and cone. The phenomena. The radial variable surface temperature $T_{w} = T_{\infty } + cr^{n}$, where $c$ and $n$ are kept constant. Here, $p$ is the pressure depends on radial $r$ and axial $z$ distance between the conical gaps. Based on the above presumption, the flow mechanism can be stated as^38^:

1 $$ \frac{\partial u}{{\partial r}} + \frac{u}{r} + \frac{\partial w}{{\partial z}} = 0, $$

2 $$ \rho_{hnf} \left( {u\frac{\partial u}{{\partial r}} - \frac{{v^{2} }}{r} + w\frac{\partial u}{{\partial z}}} \right) = - \frac{\partial p}{{\partial r}} + \mu_{hnf} \left( {\frac{{\partial^{2} u}}{{\partial r^{2} }} + \frac{1}{r}\frac{\partial u}{{\partial r}} - \frac{u}{{r^{2} }} + \frac{{\partial^{2} u}}{{\partial z^{2} }}} \right) - \sigma_{hnf} B_{0}^{2} u, $$

3 $$ \rho_{hnf} \left( {u\frac{\partial v}{{\partial r}} + \frac{uv}{r} + w\frac{\partial v}{{\partial z}}} \right) = \mu_{hnf} \left( {\frac{{\partial^{2} v}}{{\partial r^{2} }} + \frac{1}{r}\frac{\partial v}{{\partial r}} - \frac{v}{{r^{2} }} + \frac{{\partial^{2} v}}{{\partial z^{2} }}} \right) - \sigma_{hnf} B_{0}^{2} v, $$

4 $$ \rho_{hnf} \left( {u\frac{\partial w}{{\partial r}} + w\frac{\partial w}{{\partial z}}} \right) = - \frac{\partial p}{{\partial z}} + \mu_{hnf} \left( {\frac{{\partial^{2} w}}{{\partial r^{2} }} + \frac{1}{r}\frac{\partial w}{{\partial r}} + \frac{{\partial^{2} w}}{{\partial z^{2} }}} \right), $$

5 $$ \left( {\rho cp} \right)_{hnf} \left( {u\frac{\partial T}{{\partial r}} + w\frac{\partial T}{{\partial z}}} \right) = k_{hnf} \frac{{\partial^{2} T}}{{\partial z^{2} }} + \mu_{hnf} \left[ {\left( {\frac{\partial u}{{\partial z}}} \right)^{2} + \left( {\frac{\partial v}{{\partial z}}} \right)^{2} } \right] + \sigma_{hnf} B_{0}^{2} \left( {u^{2} + v^{2} } \right), $$

6 $$ \left( {u\frac{\partial C}{{\partial r}} + w\frac{\partial C}{{\partial z}}} \right) = D_{hnf} \frac{{\partial^{2} C}}{{\partial z^{2} }}, $$

7 $$ \left( {w\frac{{\partial \tilde{N}}}{\partial z} + \tilde{w}\frac{{\partial \tilde{N}}}{\partial z} + \tilde{N}\frac{{\partial \tilde{w}}}{\partial z}} \right) = D_{n} \frac{{\partial^{2} \tilde{N}}}{{\partial z^{2} }}. $$

Here, Eq. (1) is the continuity equation, Eqs. (2)–(4) are the momentum equations along *u*, *v* and *w* direction. Where, Eq. (5) represents energy transition rate, Eqs. (6) and (7) introduced concentration and motile microorganism profiles respectively. Here, $\tilde{N}$, *D*_*n,*_* Wc*, $B_{0}$ and $p$ is the motile microorganism’s density, microorganism diffusion, floating velocity of cell, magnetic strength and pressure. While $k_{hnf}$,$\nu_{hnf}$,$\rho_{hnf}$,$\mu_{hnf}$, $\left( {\rho c_{p} } \right)_{hnf}$ and $\left( {u,v,w} \right)$ is the thermal conductivity, kinematic viscosity, density, dynamic viscosity, electrical conductivity, heat capacitance and velocity terms along $r,\varphi ,z$ direction.

The boundary conditions are:

8 $$ \begin{gathered} u = 0, \, v = \omega r,\, \, w = 0,\,\,\,C = C_{w} , \, \tilde{N} = \tilde{N}_{w} ,\,\,\,T = T_{w} \,\,{\text{at}}\,\,\,{\text{z = 0}} \hfill \\ u = 0, \, v = \Omega r, \, w = 0,\,\,\,C = C_{\infty } ,\,\,\tilde{N} = \tilde{N}_{\infty } ,\,\,\,T = T_{\infty } \,\,{\text{at}}\,\,\,z = r\tan \gamma \hfill \\ \end{gathered} $$

Here $\gamma$ specified the gap angle between the cone and disk.

### Similarity conversion

We adopt the following similarity transformation^39^:

9 $$ u = \frac{{\upsilon_{f} }}{r}f\left( \eta \right){,}\,\,\,v = \frac{{\upsilon_{f} }}{r}g\left( \eta \right),\,\,w = \frac{{\upsilon_{f} }}{r}h\left( \eta \right), \, p{ = }\frac{{\rho \upsilon_{f}^{2} }}{{r^{2} }}P,\,\,\,\eta = \frac{z}{r},\,\,\,\Theta = \frac{{T - T_{\infty } }}{{T_{w} - T_{\infty } }},\,\,\mathchar'26\mkern-10mu\lambda = \frac{{N - N_{\infty } }}{{N_{w} - N_{\infty } }},\,\,\,\Phi = \frac{{C - C_{\infty } }}{{C_{w} - C_{\infty } }}. $$

Using Eqs. (8) in Eqs. (2–5), we get:

10 $$ h^{\prime} = \eta f^{\prime}, $$

11 $$ \left( {1 + \eta^{2} } \right)f^{\prime\prime} = - 3\eta f^{\prime} - D_{1} A_{1} \left( {\eta ff^{\prime} - hf^{\prime} + f^{2} - g^{2} } \right) - D_{1} \left( {2p + \eta p^{\prime} - Mf} \right), $$

12 $$ (1 + \eta^{2} )g^{\prime\prime} = D_{1} \,B_{1} \left( {\eta fg^{\prime} - hg^{\prime}} \right) + D_{1} gM - 3\eta g^{\prime}, $$

13 $$ \left( {1 + \eta^{2} } \right)h^{\prime\prime} = - 3\eta h^{\prime} - D_{1} A_{1} \left( {\eta fh^{\prime} - hh^{\prime} + h + fh} \right) + D_{1} p^{\prime}, $$

14 $$ \begin{gathered} \frac{{k_{hnf} }}{{k_{nf} }}\left( {\left( {1 + \eta^{2} } \right)\Theta^{\prime\prime} = - \eta \left( {1 - 2n} \right)\Theta^{\prime} + n^{2} \Theta } \right) - C_{1} \,Pr\left( {\eta f\Theta^{\prime} - h\Theta^{\prime} - nf\Theta } \right) - \left( {(f^{\prime})^{2} + (g^{\prime})^{2} } \right)Ec \hfill \\ - \frac{MEc}{{D_{1} }}\left( {f^{2} + g^{2} } \right), \hfill \\ \end{gathered} $$

15 $$ \left( {1 + \eta^{2} } \right)D_{1} \,\Phi^{\prime\prime} = Sc\left( {f\Phi^{\prime}} \right), $$

16 $$ \mathchar'26\mkern-10mu\lambda^{\prime\prime} = - {\text{Re}} \left( {2Scf\mathchar'26\mkern-10mu\lambda^{\prime} + Pe\left( {\mathchar'26\mkern-10mu\lambda^{\prime}\Phi^{\prime} - \mathchar'26\mkern-10mu\lambda \Phi^{\prime\prime}} \right)} \right). $$

where,

17 $$ \begin{gathered} A_{1} = \left[ {\left( {1 - \phi_{MgO} } \right)\left( {1 - \left( {1 - \frac{{\rho_{Ag} }}{{\rho_{f} }}} \right)\phi_{Ag} } \right) + \phi_{MgO} \left( {\frac{{\rho_{MgO} }}{{\rho_{f} }}} \right)} \right],\,B_{1} = \left[ \begin{gathered} \left( {1 - \phi_{Cu} } \right)\left( {1 - \left( {1 - \frac{{\rho_{Ag} }}{{\rho_{f} }}} \right)\phi_{Ag} } \right) \hfill \\ + \phi_{MgO} \left( {\frac{{\rho_{MgO} }}{{\rho_{f} }}} \right) \hfill \\ \end{gathered} \right], \hfill \\ C_{1} = \left[ {(1 - \phi_{MgO} )\left( {1 - \left( {1 - \frac{{(\rho C_{p} )_{Ag} }}{{(C_{p} \rho )_{f} }}\phi_{Ag} } \right) + \frac{{(\rho C_{p} )_{MgO} }}{{(C_{p} \rho )_{f} }}\phi_{MgO} } \right)} \right],\,D_{1} = \left( {1 - \phi_{Ag} } \right)^{2.5} \left( {1 - \phi_{MgO} } \right)^{2.5} . \hfill \\ \end{gathered} $$

The modified conditions are:

18 $$ \begin{gathered} f(0) = 0,\,\,\,g(0) = {\text{Re}}_{\omega } ,\,\,h(0) = 0,\,\,\,\Theta (0) = 1,\,\,\,\,\Phi (0) = 1,\,\,\,\mathchar'26\mkern-10mu\lambda (0) = 1, \hfill \\ f(\eta_{0} ) = 0,\,\,g(\eta_{0} ) = {\text{Re}}_{\Omega } ,\,\,\,h(\eta_{0} ) = 0,\,\,\Theta (\eta_{0} ) = 0,\,\,\Phi (\eta_{0} ) = 0,\,\,\mathchar'26\mkern-10mu\lambda (\eta_{0} ) = 0. \hfill \\ \end{gathered} $$

The volumetric fraction of *Ag* and *MgO* are demonstrated through $\phi_{Ag}$ and $\phi_{MgO}$. While $k_{hnf}$ $k_{f}$ is the thermal conductivity of hybrid nanoliquid and water.

### Thermo-physical properties

The following are the thermal properties of hybrid nanofluid and water are^42^:

**Table:** 

$\upsilon_{hnf} = \frac{{\mu_{hnf} }}{{\rho_{hnf} }},$	$\mu_{hnf} = \frac{{\mu_{f} }}{{(1 - \phi_{Ag} )^{5/2} (1 - \phi_{MgO} )^{5/2} }},$
$$\frac{{(\rho )_{hnf} }}{{(\rho )_{f} }} = \left( {1 - \phi_{MgO} } \right)\left( {1 - \left( {1 - \frac{{\rho_{Ag} }}{{\rho_{f} }}} \right)\phi_{Ag} } \right) + \phi_{MgO} \left( {\frac{{\rho_{MgO} }}{{\rho_{f} }}} \right),$$	
$$\frac{{(\rho C_{p} )_{hnf} }}{{(\rho C_{p} )_{f} }} = (1 - \phi_{MgO} )\left\{ {1 - \left( {1 - \frac{{(\rho C_{p} )_{Ag} }}{{(\rho C_{p} )_{f} }}} \right)\phi_{A} } \right\} + \frac{{(\rho C_{p} )_{MgO} }}{{(\rho C_{p} )_{f} }}\phi_{MgO} ,$$	
$$\frac{{k_{hnf} }}{{k_{nf} }} = \frac{{k_{MgO} + 2k_{nf} - 2\phi_{MgO} (k_{nf} - k_{MgO} )}}{{k_{MgO} + 2k_{nf} + \phi_{Cu} (k_{nf} - k_{MgO} )}},$$	
$$\,\,\frac{{k_{nf} }}{{k_{f} }} = \frac{{k_{Ag} + 2k_{f} - 2\phi_{Ag} (k_{f} - k_{Ag} )}}{{k_{Ag} + 2k_{f} + \phi_{Ag} (k_{f} - k_{Ag} )}}.$$	

The dimensionless form of cone and disk are rebound as:

19 $$ Nu_{d} = - \frac{{k_{hnf} }}{{k_{nf} }}\Theta^{\prime}(0),Nu_{c} = - \frac{{k_{hnf} }}{{k_{nf} }}\Theta^{\prime}(\eta_{0} )\,. $$

## Parametric continuation method

The basic idea of application of PCM method, to the system of ODE (10)–(17) with the boundary condition (18), is presented with the following steps^43^:

**Step 1:** We put forward the following submission to transfigure the system of BVP to the first-order ODE:

20 $$ \left. \begin{gathered} \ell_{1} = f(\eta ),\,\,\ell_{2} = f^{\prime}(\eta ),\,\,\,\ell_{3} = g(\eta ),\,\,\ell_{4} = g^{\prime}(\eta ),\,\,\ell_{5} = h(\eta ),\,\,\ell_{6} = h^{\prime}(\eta ),\,\,\,\ell_{7} = p\left( \eta \right),\, \hfill \\ \ell_{8} = \Theta (\eta ),\,\,\,\,\ell_{9} = \Theta^{\prime}(\eta ),\,\,\,\,\ell_{10} = \Phi (\eta ),\,\,\,\,\ell_{11} = \Phi^{\prime}(\eta ),\,\,\,\,\ell_{12} = \mathchar'26\mkern-10mu\lambda (\eta ),\,\,\,\,\ell_{13} = \mathchar'26\mkern-10mu\lambda^{\prime}(\eta ). \hfill \\ \end{gathered} \right\} $$

Using Eq. (20) in the BVP (10–16) and (18), we get:

21 $$ \ell^{\prime}_{5} = \eta \ell_{2} , $$

22 $$ \left( {1 + \eta^{2} } \right)\ell^{\prime}_{2} = - 3\eta \ell_{2} - A_{1} D_{1} \left( {\eta \ell_{1} \ell_{2} - \ell_{5} \ell_{2} + \ell_{1}^{2} - \ell_{3}^{2} } \right) - D_{1} \left( {2\ell_{7} + \eta \ell_{7} + \eta \ell^{\prime}_{7} - M\ell_{1} } \right), $$

23 $$ \left( {1 + \eta^{2} } \right)\ell^{\prime}_{4} = D_{1} B_{1} \left( {\eta \ell_{1} \ell_{4} - \ell_{5} \ell_{4} } \right) + D_{1} \ell_{3} M - 3\eta \ell_{4} , $$

24 $$ \left( {1 + \eta^{2} } \right)\ell^{\prime}_{6} = - 3\eta \ell_{6} - D_{1} A_{1} \left( {\eta \ell_{1} \ell_{6} - \ell_{5} \ell_{6} + \ell_{5} + \ell_{1} \ell_{5} + D_{1} \ell^{\prime}_{7} } \right), $$

25 $$ \left( {1 + \eta^{2} } \right)\ell^{\prime}_{9} = - \left( {\eta \left( {1 - 2n} \right)\ell_{9} + n^{2} \ell_{8} } \right) - C_{1} \,Pr\left( {\eta \ell_{1} \ell_{8} - n\ell_{1} \ell_{8} - \ell_{5} \ell_{9} } \right) - Ec\left( {\ell_{2}^{2} + \ell_{4}^{2} } \right) - \frac{MEc}{{D_{1} }}\left( {\ell_{1}^{2} + \ell_{3}^{2} } \right), $$

26 $$ \left( {1 + \eta^{2} } \right)D_{1} \ell^{\prime}_{11} = Sc\left( {\ell_{1} \ell_{11} } \right), $$

27 $$ \ell^{\prime}_{13} = - Re\left( {2Sc\ell_{1} \ell_{12} + Pe\left( {\ell_{13} \ell_{11} - \ell_{12} \ell^{\prime}_{11} } \right)} \right). $$

the reduced boundary conditions are:

28 $$ \begin{gathered} \ell_{1} \left( 0 \right) = \ell_{5} \left( 0 \right) = 0,\,\,\,\,\ell_{3} \left( 0 \right) = Re_{\omega } ,\,\,\,\ell_{8} \left( 0 \right) = 1,\,\,\,\ell_{10} \left( 0 \right) = 1,\,\,\ell_{12} \left( 0 \right) = 1, \hfill \\ \ell_{1} \left( {\eta_{0} } \right) = \ell_{5} \left( {\eta_{0} } \right) = 0,\,\,\,\ell_{3} \left( {\eta_{0} } \right) = Re_{\omega } ,\,\,\ell_{8} \left( {\eta_{0} } \right) = 0,\,\,\,\ell_{10} \left( {\eta_{0} } \right) = 0,\,\,\ell_{12} \left( {\eta_{0} } \right) = 0. \hfill \\ \end{gathered} $$

**Step 2**: Introducing parameter *p* in Eqs. (25)–(30) as follow:

29 $$ \ell^{\prime}_{5} = \eta \ell_{2} + \ell_{5} - \left( {\ell_{5} - 1} \right)p, $$

30 $$ \left( {1 + \eta^{2} } \right)\ell^{\prime}_{2} = - 3\eta \left( {\ell_{2} - 1} \right)p - A_{1} D_{1} \left( {\eta \ell_{1} \ell_{2} - \ell_{5} \ell_{2} + \ell_{1}^{2} - \ell_{3}^{2} } \right) - D_{1} \left( {2\ell_{7} + \eta \ell_{7} + \eta \ell^{\prime}_{7} - M\ell_{1} } \right), $$

31 $$ \left( {1 + \eta^{2} } \right)\ell^{\prime}_{4} = D_{1} B_{1} \left( {\eta \ell_{1} \left( {\ell_{4} - 1} \right)p - \ell_{5} \ell_{4} } \right) + D_{1} \ell_{3} M - 3\eta \ell_{4} , $$

32 $$ \left( {1 + \eta^{2} } \right)\ell^{\prime}_{6} = - 3\eta \ell_{6} - D_{1} A_{1} \left( {\eta \ell_{1} \ell_{6} - \ell_{5} \ell_{6} + \ell_{5} + \ell_{1} \ell_{5} + D_{1} \ell^{\prime}_{7} } \right), $$

33 $$ \begin{aligned} \left( {1 + \eta^{2} } \right)\ell^{\prime}_{9} & = - \left( {\eta \left( {1 - 2n} \right)\left( {\ell_{9} - 1} \right)p + n^{2} \ell_{8} } \right) \\ & \quad - C_{1} \,Pr\left( {\eta \ell_{1} \ell_{8} - n\ell_{1} \ell_{8} - \ell_{5} \ell_{9} } \right) - Ec\left( {\ell_{2}^{2} + \ell_{4}^{2} } \right) - \frac{MEc}{{D_{1} }}\left( {\ell_{1}^{2} + \ell_{3}^{2} } \right), \\ \end{aligned} $$

34 $$ \left( {1 + \eta^{2} } \right)D_{1} \ell^{\prime}_{11} = Sc\left( {\ell_{1} \left( {\ell_{11} - 1} \right)p} \right), $$

35 $$ \ell^{\prime}_{13} = - Re\left( {2Sc\ell_{1} \ell_{12} + Pe\left( {\left( {\ell_{13} - 1} \right)p\ell_{11} - \ell_{12} \ell^{\prime}_{11} } \right)} \right). $$

**Step 3**: Differentiating by parameter ‘p’

While differentiating Eqs. (29–35) for parameter p, come at the following system in terms of parameter p:

36 $$ V^{\prime} = AV + R, $$

where R and A are the remainder and coefficient matrix respectively.

37 $$ \frac{{d\zeta_{i} }}{d\tau } $$

where *i* = 1*,* 2*, …*11*.*

**Step 4**: Use the superposition approach to each problem and characterize the Cauchy problem

38 $$ V = aU + W, $$

For each term, resolve the Cauchy problems below.

39 $$ U^{\prime} = aU, $$

40 $$ W^{\prime} = AW + R, $$

We obtained the estimated solution by putting Eq. (38) in Eq. (36).

41 $$ (aU + W)^{\prime} = A(aU + W) + R, $$

**Step 5**: Solving the Cauchy problems

This study employs a numerical implicit methodology, which is detailed below.

42 $$ \frac{{U^{i + 1} - U^{i} }}{\Delta \eta } = AU^{i + 1} ,\,\,or\,\,\,\,U^{i + 1} (I - \Delta \eta A) = U^{i} , $$

43 $$ \frac{{W^{i + 1} - W^{i} }}{\Delta \eta } = AW^{i + 1} ,\,\,or\,\,\,\,W^{i + 1} (I - \Delta \eta A) = W^{i} , $$

we get the iterative form of the solution.

44 $$ U^{i + 1} = (I - \Delta \eta A)^{ - 1} U^{i} , $$

45 $$ W^{i + 1} = (I - \Delta \eta A)^{ - 1} (W^{i} + \Delta \eta R). $$

## Result and discussion

The goal of this part is to learn about the effects of velocity, temperature, mass, and motile microorganism distributions under the effect of multiple fundamental factors. The flow mechanics of a circulating cone and disc is observed in Fig. 1. Table 1 addressed the numerical properties of silver, magnesium oxide and water. The four different cases are discussed in detail between cone and disk. Case 1 elaborated that the disk is spinning while the cone is fixed. Case 2 revealed that the cone is spinning, while the disk is fixed. Case 3 & 4 highlighted that the disk and cone are co-rotating or counter-rotating respectively. The following observations have been noticed:

**Table 1:** The numerical properties of silver, magnesium oxide and water^40,41^.

	$\rho (kg/m^{3} )$	$C_{p} (j/kgK)$	$k(W/mK)$	$\beta \times 10^{5} \left( {K^{ - 1} } \right)$	*Pr*
Pure water	997.1	4179	0.613	21	6.2
Magnesium oxide	3560	955	45	1.80	
Silver	10,500	235	429	1.89	

### Axial velocity profile

**Figure 2:**
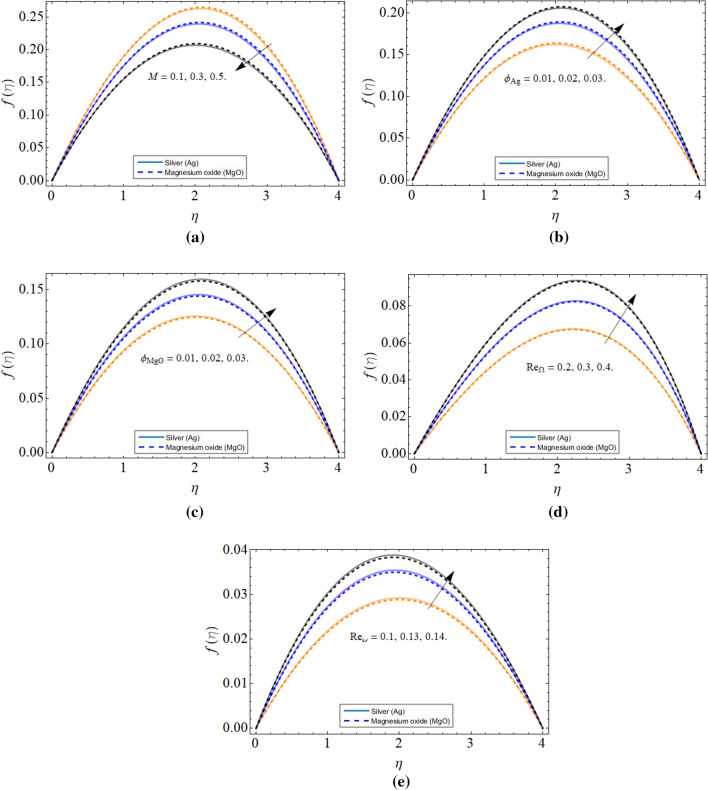
The behavior of axial velocity profile $f\left( \eta \right)$ versus (**a**) magnetic field *M* (**b**) volume friction of silver $\phi_{Ag}$ (**c**) volume friction of magnesium oxide $\phi_{MgO}$ (**d**) cone angular velocity $Re_{\Omega }$ (**e**) disk angular velocity $Re_{\omega }$.

Figure 2a–e revealed the behavior of axial velocity $f\left( \eta \right)$ profile versus magnetic field *M*, volume friction of silver $\phi_{Ag}$, volume friction of magnesium oxide $\phi_{MgO}$, cone angular velocity $Re_{\Omega }$ and disk angular velocity $Re_{\omega }$ respectively. The resistive force generated due to the consequences of the magnetic field declines the axial velocity as shown in Fig. 2a. Figure 2b,c manifested that the velocity profile enhances with the increment of both silver *Ag* and magnesium oxide MgO nanomaterials because the specific heat capacity of MgO and *Ag* nanoparticles is much less than water, that’s why the rising thermal energy inside the hybrid nanoliquid also causes the rises in fluid velocity. Figure 2d,e displayed that the velocity profile $f\left( \eta \right)$ significantly boosts with the growing values of both cone $\Omega$ and disk $\omega$ rotation. Physically, the improvement in both device's angular velocity encourages the fluid particles to move fast, which causes the inclination of axial velocity $f\left( \eta \right)$ across disk and cone.

### Radial velocity profile

**Figure 3:**
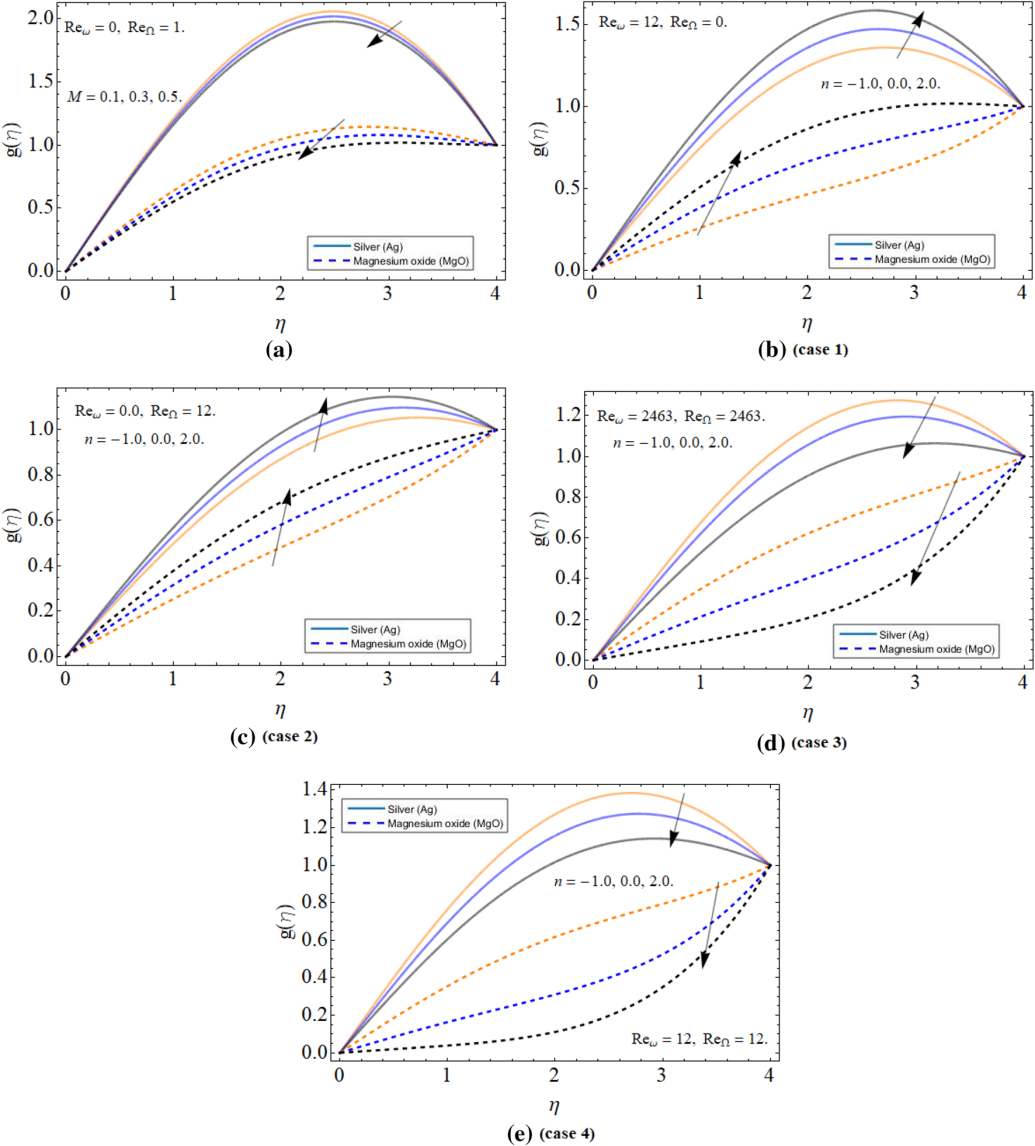
The behavior of radial velocity profile $g\left( \eta \right)$ versus (**a**) magnetic field *M* (**b**) disk rotation (**c**) cone rotation (**d**) both disk and cone co-rotation (**e**) both disk and cone counter rotation.

Figure 3a–e highlighted the behavior of radial velocity profile $g\left( \eta \right)$ versus magnetic field and four different cases of rotation and counter-rotation of both cone and disk respectively. The magnetic effect is also reducing the radial velocity, while keeping disk stationery and cone moving as illustrated in Fig. 3a. Figure 3b,c highlighted the two cases (1 & 2) and pointed that the radial velocity increases in both cases. Physically, the increasing velocity of both apparatuses excited the fluid particles, which become the reason for the enhancement of the radial velocity profile $g\left( \eta \right)$. Figure 3d,e connived cases 3 & 4 and shows that the velocity distribution reduces in both cases. The counter-rotation of the disk and cone generated resistance to the flow field, which decline the velocity profile.

### Tangential velocity profile

**Figure 4:**
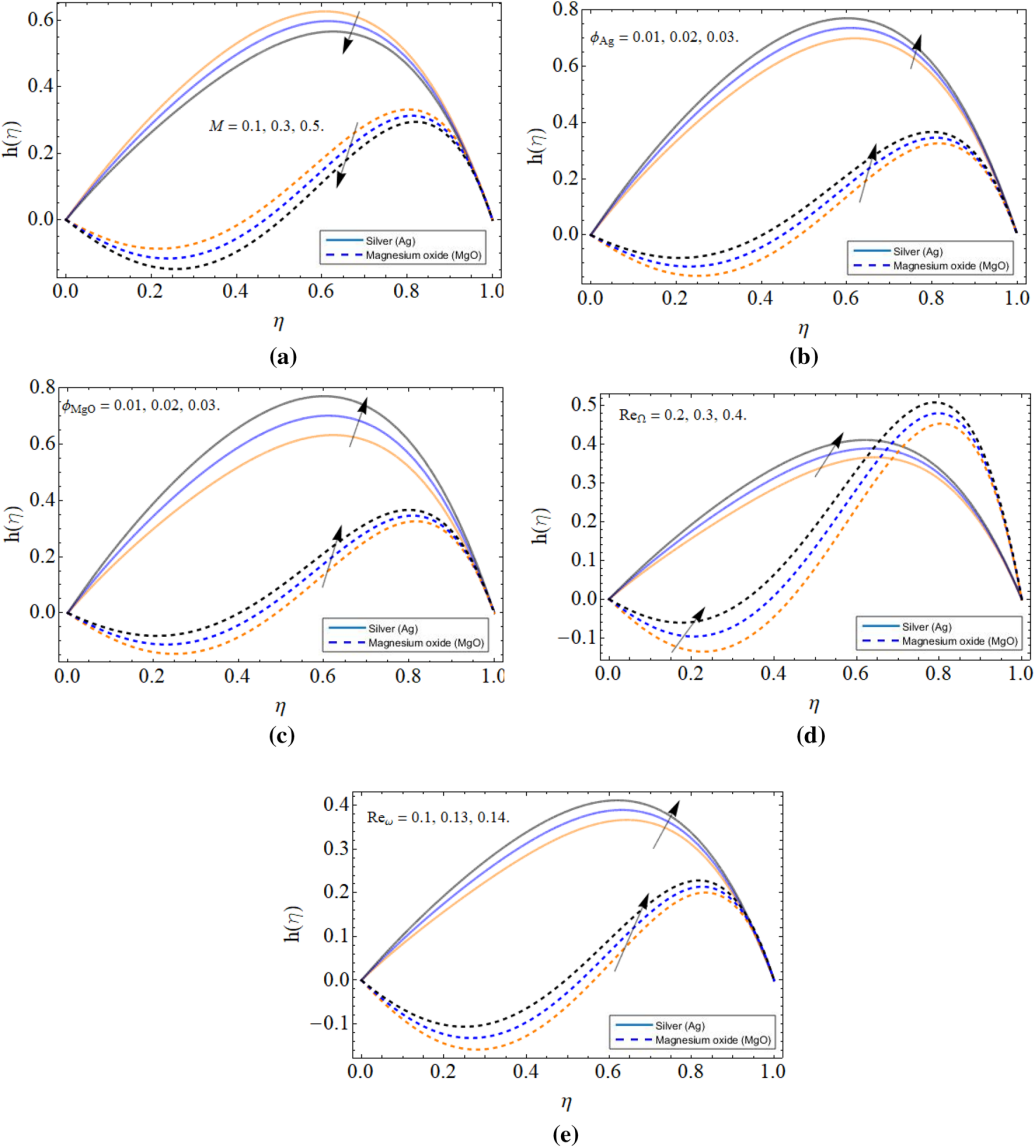
The behavior of tangential velocity profile $h\left( \eta \right)$ versus (**a**) magnetic field *M* (**b**) volume friction of silver $\phi_{Ag}$ (**c**) volume friction of magnesium oxide $\phi_{MgO}$ (**d**) cone angular velocity $Re_{\Omega }$ (**e**) disk angular velocity $Re_{\omega }$.

Figure 4a–e elaborated the nature of tangential velocity profile $h\left( \eta \right)$ versus magnetic field *M*, volume friction of silver $\phi_{Ag}$, volume friction of magnesium oxide $\phi_{MgO}$, cone angular velocity $Re_{\Omega }$ and disk angular velocity $Re_{\omega }$ respectively. The tangential velocity of the fluid is significantly reducing with the influence of the magnetic field as shown in Fig. 4a. Figure 4b–e illustrated that the tangential velocity $h\left( \eta \right)$ profile boosts with the rising quantity of nanoparticles (*Ag* & MgO) and both disk $Re_{\omega }$ and cone $Re_{\Omega }$ increasing rotation respectively.

### Thermal energy profile

**Figure 5:**
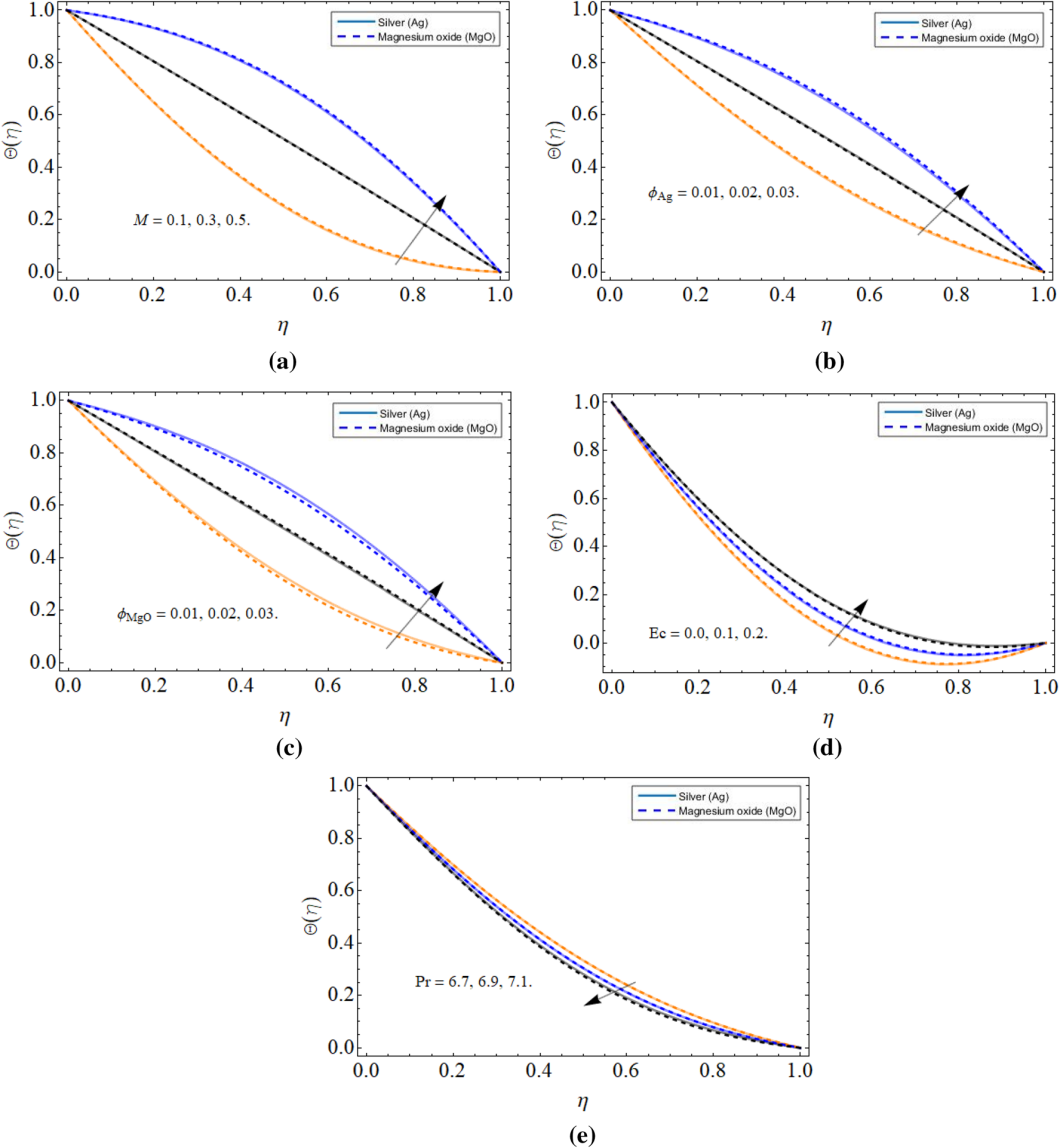
The behavior of thermal energy profile $\Theta \left( \eta \right)$ versus (**a**) magnetic field *M* (**b**) volume friction of silver $\phi_{Ag}$ (**c**) volume friction of magnesium oxide $\phi_{MgO}$ (**d**) Eckert number *Ec* (**e**) Prandtl number *Pr*.

Figure 5a–e displayed the characteristics of thermal energy profile $\Theta \left( \eta \right)$ versus magnetic field *M*, volume friction of silver $\phi_{Ag}$, volume friction of magnesium oxide $\phi_{MgO}$, Eckert number *Ec* and Prandtl number *Pr* respectively. Figure 5a–d communicated that the thermal energy profile remarkably upgraded with the magnetic effect, the addition of nanoparticulated in base fluid and Eckert number respectively. As we have discussed in Fig. 2b,c that the growing credit of nanoparticles diminishes the average specific heat capacity of base fluid, that’s why such a scenario has been observed in Fig. 5b,c. Similarly, the dissipation energy is added to fluid internal energy and enhances its thermal energy $\Theta \left( \eta \right)$ distribution as exposed in Fig. 5d. The higher Prandtl fluid *Pr* has always less thermal diffusivity, that’s why the Prandtl effect decrease fluid temperature $\Theta \left( \eta \right)$.

**Figure 6:**
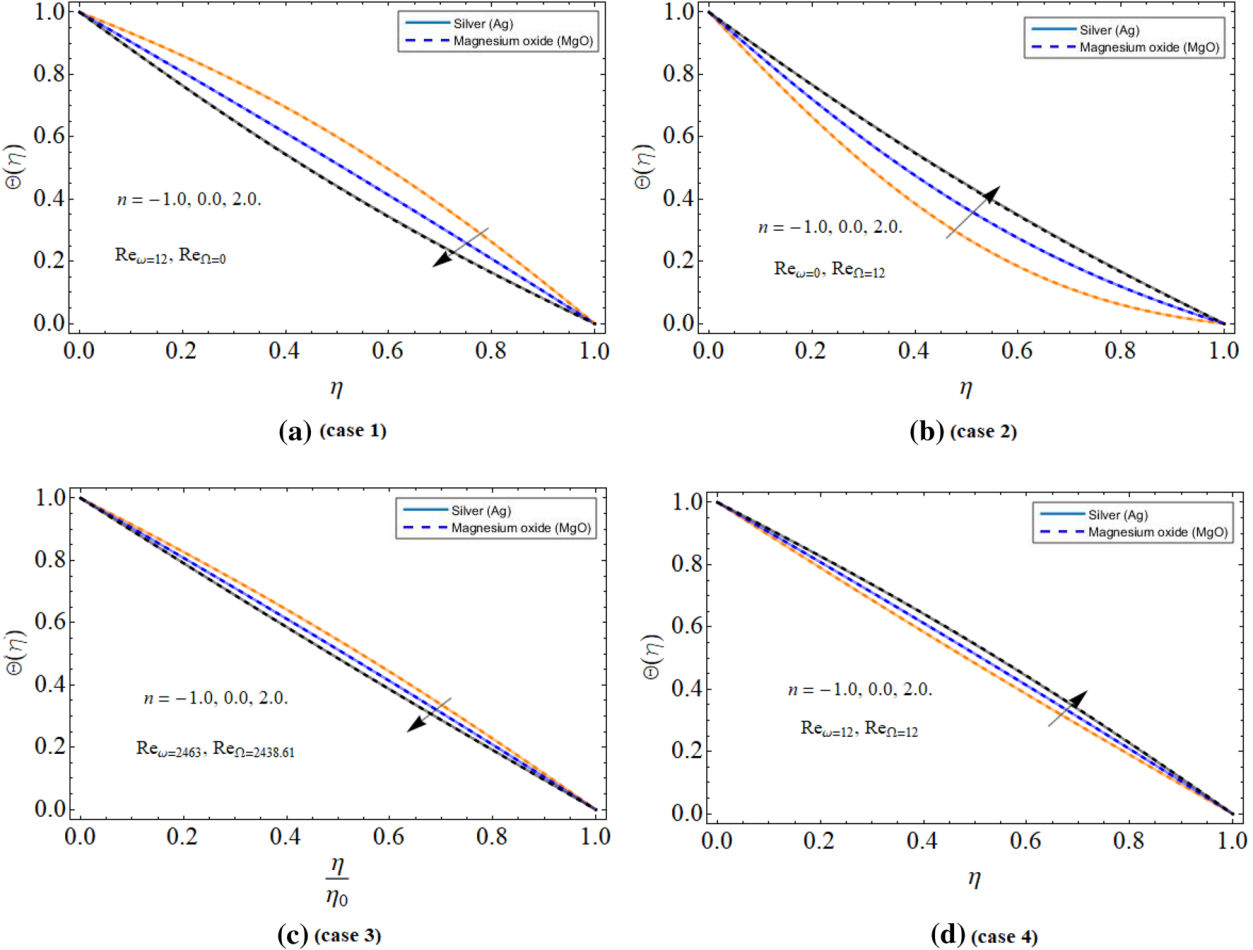
The behavior of thermal energy profile $\Theta \left( \eta \right)$ versus (**a**) disk rotation (**b**) cone rotation (**c**) both disk and cone co-rotation (**d**) both disk and cone counter-rotation.

Figure 6a–d spotted the four different cases between the cone and disk. The rotation of the disk, while keeping the cone fixed reduces the energy profile as appeared in Fig. 6a. An opposite scenario has been observed while keeping the disk fixed and spinning cone Fig. 6b. Figure 6c,d revealed that the energy profile reduces with the co-rotation of disk and cone, while enhances with counter-rotation because the opposite direction motion generates resistive force, which encourages fluid temperature $\Theta \left( \eta \right)$ as scrutinized in Fig. 6d.

### Concentration and motile microorganism profile

**Figure 7:**
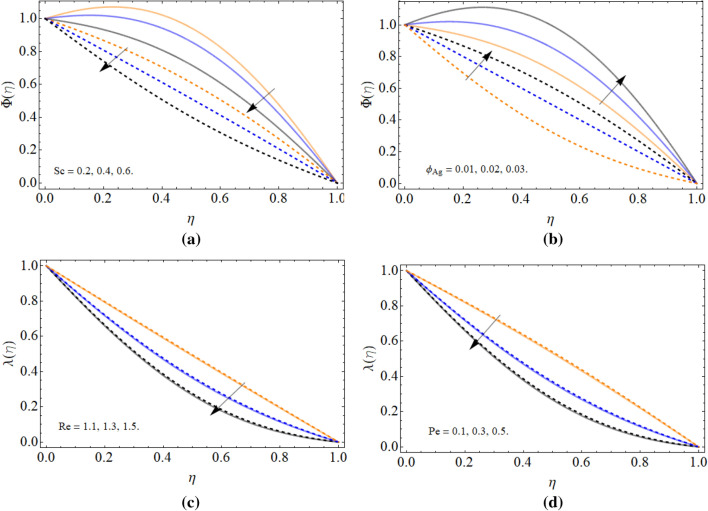
The behavior of mass transfer profile $\Phi \left( \eta \right)$ and motile microorganism $\mathchar'26\mkern-10mu\lambda \left( \eta \right)$ versus (**a**) Schmidt number *Sc* (**b**) volume friction of silver $\phi_{Ag}$ (**c**) Reynold number *Re* (**d**) Peclet number *Pe*.

Figure 7a–d manifested the behavior of mass transfer profile $\Phi \left( \eta \right)$ and motile microorganism $\mathchar'26\mkern-10mu\lambda \left( \eta \right)$ versus Schmidt number *Sc*, volume friction of silver $\phi_{Ag}$, Reynold number *Re* and Peclet number *Pe* respectively. The Schmidt number upshot diminished the mass transition rate as shown in Fig. 7a. Physically, the molecular diffusion lowers, and kinetic viscosity rises with Schmidt number, that’s why such phenomena have been observed. An opposite scenario is observed against silver nanoparticles $\phi_{Ag}$ that the mass transference enhances with the upshot of silver nanoparticles as elaborated through Fig. 7b. Figure 7c,d illustrated that the bioconvection Reynold number *Re* and Peclet number *Pe* declines the motile microorganism $\mathchar'26\mkern-10mu\lambda \left( \eta \right)$ profile. Because the density of microorganism decreases with the upshot of both Reynold number *Re* and Peclet number.

Tables 2, 3, 4 and 5 exhibits the numerical results of the Nusselt number $- \Theta^{\prime}\left( 0 \right)$ and Sherwood number $- \Phi^{\prime}\left( 0 \right)$ at both, the disc surface and cone surface for the $Ag$ and $Ag + MgO$ respectively. While Table 6 revealed comparative analysis for the validation of the results between PCM and bvp4c numerical Matlab package.

**Table 2:** Numerical outputs of Nusselt number $- \Theta^{\prime}\left( 0 \right)$ at the surface of disc.

$\Pr$	$$\begin{gathered} - \Theta^{\prime}(0)\,Ag \hfill \\ \phi_{Ag} = 0.02 \hfill \\ \end{gathered}$$	$$\begin{gathered} - \Theta^{\prime}(0)\,Ag \hfill \\ \phi_{Ag} = 0.04 \hfill \\ \end{gathered}$$	$$\begin{gathered} - \Theta^{\prime}(0)\,Ag + MgO \hfill \\ \phi_{Ag} = \phi_{MgO} = 0.02 \hfill \\ \end{gathered}$$	$$\begin{gathered} - \Theta^{\prime}(0)\,Ag + MgO \hfill \\ \phi_{Ag} = \phi_{MgO} = 0.04 \hfill \\ \end{gathered}$$
6.4	0.976487	0.979312	0.976945	0.981377
6.5	0.975697	0.979587	0.976274	0.979668
6.6	0.974928	0.978862	0.975492	0.969958

**Table 3:** Numerical outputs of Nusselt number $- \Theta^{\prime}\left( 1 \right)$ at the surface of cone.

$\Pr$	$$\begin{gathered} - \Theta^{\prime}(1)\,Ag \hfill \\ \phi_{Ag} = 0.01 \hfill \\ \end{gathered}$$	$$\begin{gathered} - \Theta^{\prime}(1)\,Ag \hfill \\ \phi_{Ag} = 0.02 \hfill \\ \end{gathered}$$	$$\begin{gathered} - \Theta^{\prime}(1)\,Ag + MgO \hfill \\ \phi_{Ag} = \phi_{MgO} = 0.01 \hfill \\ \end{gathered}$$	$$\begin{gathered} - \Theta^{\prime}(1)\,Ag + MgO \hfill \\ \phi_{Ag} = \phi_{MgO} = 0.02 \hfill \\ \end{gathered}$$
6.4	1.14573	1.24956	2.77864	2.78345
6.5	1.14487	1.23471	2.76196	2.77784
6.6	1.14367	1.22182	2.75845	2.76399

**Table 4:** Numerical outputs of Sherwood number $- \Phi^{\prime}\left( 0 \right)$ at the surface of disc.

$Sc$	$$\begin{gathered} - \Phi^{\prime}(0)\,Ag \hfill \\ \phi_{Ag} = 0.02 \hfill \\ \end{gathered}$$	$$\begin{gathered} - \Phi^{\prime}(0)\,Ag \hfill \\ \phi_{Ag} = 0.04 \hfill \\ \end{gathered}$$	$$\begin{gathered} - \Phi^{\prime}(0)\,Ag + MgO \hfill \\ \phi_{Ag} = \phi_{MgO} = 0.02 \hfill \\ \end{gathered}$$	$$\begin{gathered} - \Phi^{\prime}(0)\,Ag + MgO \hfill \\ \phi_{Ag} = \phi_{MgO} = 0.04 \hfill \\ \end{gathered}$$
0.2	0.896487	0.899312	0.896945	0.891377
0.3	0.895697	0.899587	0.896274	0.899668
0.4	0.894928	0.898862	0.895492	0.899958

**Table 5:** Numerical outputs of Sherwood number $- \Phi^{\prime}\left( 1 \right)$ at the surface of cone.

$Sc$	$$\begin{gathered} - \Phi^{\prime}(1)\,Ag \hfill \\ \phi_{Ag} = 0.01 \hfill \\ \end{gathered}$$	$$\begin{gathered} - \Phi^{\prime}(1)\,Ag \hfill \\ \phi_{Ag} = 0.02 \hfill \\ \end{gathered}$$	$$\begin{gathered} - \Phi^{\prime}(1)\,Ag + MgO \hfill \\ \phi_{Ag} = \phi_{MgO} = 0.01 \hfill \\ \end{gathered}$$	$$\begin{gathered} - \Phi^{\prime}(1)\,Ag + MgO \hfill \\ \phi_{Ag} = \phi_{MgO} = 0.02 \hfill \\ \end{gathered}$$
0.2	1.04573	1.04956	2.57864	2.58345
0.3	1.04487	1.03471	2.56196	2.57784
0.4	1.04367	1.02182	2.55845	2.56399

**Table 6:** PCM and bvp4c methods comparison for $Ag$: when $\phi_{Ag} = 0.04,\,\,\phi_{MgO} = 0.02,\,\,Pr = 6.3,\,Re_{\Omega } = 0.13,\,\,Re_{\omega } = 0.3.$

No.	PCM	bvp4c	${\text{Absolute}}\,{\text{Error}}$
1	$1.63 \times 10^{ - 11}$	$6.325 \times 10^{ - 17}$	$4.3298 \times 10^{ - 14}$
2	$4.23 \times 10^{ - 12}$	$3.834 \times 10^{ - 20}$	$2.7139 \times 10^{ - 15}$
3	$4.69 \times 10^{ - 12}$	$2.8489 \times 10^{ - 19}$	$4.6682 \times 10^{ - 16}$
4	$7.429 \times 10^{ - 14}$	$8.449 \times 10^{ - 20}$	$7.6659 \times 10^{ - 17}$
5	$6.898 \times 10^{ - 14}$	$4.8378 \times 10^{ - 20}$	$8.7392 \times 10^{ - 17}$

## Conclusion

In the present study, the silver, magnesium oxide and gyrotactic microorganism-based hybrid nanofluid flow inside the conical space between disc and cone is addressed in the perspective of thermal energy stabilization. The hybrid nanofluid has been synthesized in the presence of silver *Ag* and magnesium oxide *MgO* nanoparticulate. The viscous dissipation and the magnetic field factors are introduced to the modeled equations. The numerical boundary value solver bvp4c is utilized to numerically handle the modeled problem. The following results have been observed:

- The resistive force generated due to the consequences of magnetic field declines the velocity profiles, while enhances with the increment of both silver *Ag* and magnesium oxide MgO nanomaterials.
- The velocity profile significantly boosts with the growing values of both cone $\Omega$ and disk $\omega$ rotation.
- The increasing velocity of both apparatuses (disk & cone) excited the fluid particles, which become the reason for the enhancement of velocity profiles.
- The fluid velocity boosts with the rising quantity of nanoparticles (*Ag* & MgO) and both disk $Re_{\omega }$ and cone $Re_{\Omega }$ increasing rotation.
- The thermal energy profile remarkably upgraded with the magnetic effect, the addition of nanoparticulated in base fluid and Eckert number respectively.
- The energy profile reduces with the co-rotation of disk and cone, while enhances with counter-rotation because the opposite direction motion generates resistive force, which encourages fluid temperature $\Theta \left( \eta \right)$.
- The motile microorganism profile decreases with the upshot of both Reynold number *Re* and Peclet number.

## Abbreviations

*r*: Radial distance
*c*: Constant
$Nu_{d}$: Nusselt number of cone
$$T$$: Fluid temperature (K)
$$T_{\infty }$$: Ambient temperature
*Pr*: Prandtl number
*M*: Magnetic number
${\text{Re}}_{\Omega } = r^{2} \Omega /\nu$: Local Reynold number
$$\left( {r,\varphi ,z} \right)$$: Cylindrical coordinate
$$u,v,w$$: Velocity component
$n$: Surface temperature power index
$Nu_{d}$: Nusselt number of disk
$$T_{w}$$: Surface temperature
$$p$$(Pa): Pressure
*Ec*: Eckert number
$$B_{0}$$: Magnetic field
${\text{Re}}_{\omega } = r^{2} \omega /\nu$: Local Reynold number
$$F,G,H$$: Dimensional velocity

## Greek symbols

$$\eta$$: Similarity variable
$$\nu$$: Kinematic viscosity (m^2^ s^−1^)
$\omega$: Angular velocity of disk (r s^−1^)
$$\Theta$$: Dimensionless temperature
$k_{{{\text{nf}}}}$: Thermal conductivity (Wm^−1^ K^−1^)
$\phi_{{{\text{Fe}}_{3} {\text{O}}_{4} }}$: Iron oxide volume fraction
$$k_{hnf}$$: Thermal conductivity
$$\sigma$$: Electrical conductivity (S/m)
$\rho_{hnf}$: Hybrid Nanofluid density
$$\eta_{0}$$: Cone surface
$\mu$: Dynamic viscosity (kg m^−1^ s^−1^)
$\mu_{hnf}$: Dynamic viscosity of nanofluid
$\left( {\rho c_{p} } \right)_{hnf}$: Heat capacitance of hybrid nanoliquid (kg m^2^ s^−2^ K^−1^)
$\Omega$: Angular velocity of cone (r s^−1^)
$\phi_{Cu}$: Copper volume fraction
$$\alpha$$: Thermal diffusivity (m^2^ s^−1^)
$$\gamma$$: Gap angle (°)
$\nu_{hnf}$: Nanofluid kinematic viscosity
